# Progress in Surgery

**Published:** 1900-05-19

**Authors:** 


### PROGRESS IN SURGERY.

### DISEASES OF THE RECTUM AND ANUS.

**Imperforate Rectum.**—W. P. Montgomery[1] has published an account of ten cases of this affection, in all of which he had operated. In nine the operation of colotomy was performed, in six immediately, and in the remaining three after a perineal exploration. Three of these cases, two of which survived, were sent home immediately after the operation. The other six died in hospital, and in them the conditions of the parts were carefully examined after death. The two considerations influencing the choice of operation were—(1) The size of the pelvic outlet; and (2) the general condition of the child. In none of these cases was there any bulging of the perineum during crying. The colotomy incision was on the left side, an inch below and internal to the anterior superior spine. The small gut presented in five cases, the cæcum in three, and the sigmoid in one. In the cases in which the cæcum presented it was easily brought into the wound and incised; in all the other cases there was difficulty in bringing the gut up to the level of the abdominal wall. This was apparently due to the great distension of the small gut and cæcum, not to shortness of the mesentery. The two cases which survived are now 18 months and two years old respectively. In both there is a tendency to prolapse of the bowel through the artificial anus, a tendency observed in nearly all recorded cases. It could be guarded against by passing a supporting suture through the mesentery, but the difficulty in bringing up the sigmoid loop often makes this impossible to do. A point noticed is that the tendency to peritoneal repair in these small children seems small. The gut had a very thin union with the edges of the incision, even after seven days and 17 days' interval in two cases; and in a third the sutures gave way on the twelfth day, and small intestine was prolapsed. The coils were cleaned and put back, but, though the child lived five days longer, there were no adhesions formed between these coils. The suturing of the bowel to the abdominal wall should, therefore, be very carefully performed. Montgomery gives details of the position of the end of the occluded bowel as found at the necropsies. In one case, in which an exploration had been made from the perineum, after resection of the coccyx, though the uretal pouch was only an inch from the skin it had been passed by, apparently because the exploration had been confined to the concavity of the sacrum, as it is usually advised to be. Huguier long ago suggested that in cases of imperforate rectum it is better to look for and to open the sigmoid in the right groin. Giraldés found the sigmoid on the right side 20 times in 134 cases. Montgomery has found that in 20 newly born infants it was possible that in six the sigmoid would have been missed in the operation of left inguinal colotomy, and in two of these it was actually missed and the cæcum opened. In these two cases the cæcum was distended and twisted and the sigmoid was not showing, and the acute obstruction symptoms were caused by the condition of the cæcum rather than by the rectal imperforation, and opening the cæcum was the only operation likely to benefit them. Montgomery concludes that he could only have been reasonably sure of finding the bowel from the perineum in two of the six cases he was able carefully to examine. These feeble children are not proper subjects for prolonged operations. Contrasting the perineal operation with the abdominal one as a remedy for acute symptoms we see that in the former the bowel is often difficult to find, and that opening it may fail to relieve distension of the cæcum. These are arguments in favour of routine colotomy. As a method of permanent relief it has to be remembered that the anus formed in the perineum is not a satisfactory one. In the majority of cases constant attention has to be given to prevent strictures. Montgomery says that we are probably justified in saying that if more than half an inch separates the rectal pouch from the surface a perineal anus will be a continuous source of trouble. In such cases probably an inguinal anus would be more comfortable and easily attended to.

**Non-malignant Stricture of the Rectum.**—W. Duff Bullard[2] has discussed this affection. He points out that though most observers regard syphilis as the common cause of this form of stricture, yet no convincing argument has been brought forward proving that syphilis ravages the rectum more than other portions of the alimentary canal. Bullard then proceeds to give an interesting *resumé* of the views held by numerous writers on this subject. Among more recent authors Van Buren regards the chancroid, especially when it takes on a phagedænic character, as the only mode in which any form of venereal disease is certainly known to give rise to these strictures. Mason's views may be quoted here. He reported 31 cases, all in women. He regards the disease as peculiar to females, occurring chiefly between 18 and 25 years of age. He states that anti-syphilitic remedies are useless, and that the disease being due to the chancroid, and only in very rare cases perhaps to syphilis, should be known as "chancroidal" stricture. Matthews, on the other hand, is of opinion that 99 out of every 100 cases are syphilitic. Bullard would go so far as to say that not one in 1,000 cases of stricture is caused either by chancroid or syphilis. The mucous patch of syphilis cannot be the cause, for it is an ulcerous lesion that is rarely deep, and always responds to cleanliness and anti-syphilitic treatment. Most authors look upon the ano-rectal syphiloma of Fournier as the cause of these strictures. This is supposed to be a diffuse infiltration of all the lower rectal wall, causing an hypertrophy which narrows the canal. Bullard says such a process occurs, but it is not due to syphilis, but to prolonged irritation acting in the rectum, or more frequently to external causes. He admits that the condition is most frequently seen in syphilitic subjects, but not that syphilis is a cause *per se*. The lesions that lead to the progressive narrowing are of two large classes, viz.: (1) those acting external to the bowel, producing mechanical obstructions and circulatory disturbances, resulting in involvement of the rectal wall; (2) lesions originating in the wall of the bowel itself. The former lesions occur in women much more frequently than in men, owing to the closer proximity of the organs of generation to the rectum in them. Secondary to enlargements and other diseases of the genital organs, the mucous membrane of the rectum has its nutrition disturbed, and ulceration follows, which may be initiated or aggravated by constipation, and soon stricture follows. As to symptoms, the "ribbon-shaped" stools are rarely seen in stricture unless the anus is involved, or the stricture is forced down to the anus by straining. Anything which causes spasmodic contraction, or hypertrophy of the sphincter, will produce such stools. To examine the strictures, Kelly's tubes and a forehead light are recommended. Through the tube an olive-headed bougie, with a very small metal stem, should be passed. As this passes the walls of the stricture can be well seen, and its length determined. In treatment the great indication is to cure ulceration by applications made directly to the ulcerating area. This will diminish irritation by removing infective materials, and then Nature will cause the disappearance of a large amount of the infiltration, and the bowel wall will become amenable to dilatation. It is held that the above line of treatment is better than any operation unless the patient is suffering from chronic obstruction, or the stricture is of very narrow calibre. In the latter class of cases the operation of posterior proctotomy is the most generally useful. The after-treatment of the incision is of chief importance. The object is to get a portion of the incised tissues covered with mucous membrane. Healing must be delayed, *e.g.*, by the application of the electric cautery to the granulations, and by the application of inert dusting powders, such as bismuth subnitrate, to give time for the mucous membrane to creep over the surface of the wound.

**Hæmorrhoids.**—J. W. J. Doyle[3] alludes to the importance of examination of the rectum in patients who allege that they suffer from hæmorrhoids, and to the frequency with which this is omitted by practitioners. Female patients much more readily submit to vaginal than rectal examinations, and many men have very great objections to the examination. Other diseases are frequently the exciting causes of hæmorrhoids, among them being uterine displacements, pelvic and abdominal growths, affections of the bladder, urethral stricture, and diseased prostate. The editors of the *British Physician*,[4] writing on hæmorrhoids, draw attention to the extreme prevalence of the affection, and to the frequency with which they are neglected by patients, either from natural shyness or from fear of the knife. More often than not local remedies merely ameliorate without curing. A remedy is recommended which claims not only to relieve painful symptoms, but also to exert a marked curative effect upon the rectal mucous membrane. It is iodo-resorcin sulphonate of bismuth, to which the manufacturers have given the somewhat suggestive name of anusol. It is readily decomposed, and is therefore made into suppositories immediately after being manufactured, with zinc oxide, balsam of Peru, cacao butter, and unguentum cerei. These suppositories, which should be of torpedo shape, quickly relieve pain. They are non-toxic. They act as disinfectants of the rectum, and have a decided action in arresting any inflammatory condition that may be present. Dr. Timmermann, of Hanover, has used anusol extensively, and considers it a specific for hæmorrhoids. He says it is a powerful disinfectant, deodorant, and astringent, and tends also to relieve constipation. Other writers confirm his results. L. H. Adler[5] points out that patients in nearly all cases endeavour to reduce both internal and external piles by pushing them into the rectum, and thus aggravate the latter variety. He states that external piles, whether inflamed or otherwise, can always be painlessly removed under cocaine, and that the operation does most good when they are inflamed.

**Wound of the Bladder and Rectum.**—J. R. Dodd[6] records an interesting case of this. A heavy man was hauling on a rope with another when the rope broke, and he fell backwards with the other man upon him on to the upturned end of an iron bar seven-eighths of an inch in diameter. The finger, on examination, was passed from a wound in the buttock through the rectum two inches above the anus, and on into the bladder through a rent in the prostate, impinging upon a catheter which had been introduced, Four pieces of cloth, parts of the man's clothing, were found in the rectum. The wound in the buttock was opened up into the rectum as in the operation for fistula. A catheter was tied in the bladder, the urine speedily became very offensive, fæces and flatus being mixed with it. The catheter had to be left out, owing to irritation, in three days. After that voluntary efforts at micturition resulted in expulsion of urine chiefly through the anus. The act was discouraged, the urine being drawn off by a catheter. In 18 days the urine was clear, acid, and free from smell. Two months later there was no bladder or rectal trouble, and a full-sized silver catheter could be passed without difficulty.

[1] Lancet, Feb., 1900. [2] Med. Rec., Jan., 1900. [3] New York Med. Jour., Jan., 1900. [4] The British Physician, Mar., 1900. [5] New York Med. Jour., Dec., 1899. [6] Brit. Med. Jour., Feb., 1900.

